# Evolutionary trade-offs associated with loss of PmrB function in host-adapted *Pseudomonas aeruginosa*

**DOI:** 10.1038/s41467-018-04996-x

**Published:** 2018-07-06

**Authors:** Laura Bricio-Moreno, Victoria H. Sheridan, Ian Goodhead, Stuart Armstrong, Janet K.L. Wong, Elaine M. Waters, Joscelyn Sarsby, Stavros Panagiotou, James Dunn, Adrita Chakraborty, Yongliang Fang, Karl E. Griswold, Craig Winstanley, Joanne L. Fothergill, Aras Kadioglu, Daniel R. Neill

**Affiliations:** 1 Institute of Infection and Global Health, University of Liverpool, Liverpool, L69 7BE UK; 2 School of Environment and Life Sciences, University of Salford, Salford, M5 4WT UK; 3 NIHR Health Protection Research Unit in Emerging and Zoonotic Infections, University of Liverpool, Liverpool, L69 3GL UK; 4 Institute of Integrative Biology, University of Liverpool, Liverpool, L69 7ZB UK; 5 Thayer School of Engineering, Dartmouth, Hanover, NH 03755 USA; 6 Present Address: Department of Microbiology, School of Natural Science, National University of Ireland, Galway, H91 TK33 Ireland

## Abstract

*Pseudomonas aeruginosa* colonises the upper airway of cystic fibrosis (CF) patients, providing a reservoir of host-adapted genotypes that subsequently establish chronic lung infection. We previously experimentally-evolved *P. aeruginosa* in a murine model of respiratory tract infection and observed early-acquired mutations in *pmrB*, encoding the sensor kinase of a two-component system that promoted establishment and persistence of infection. Here, using proteomics, we show downregulation of proteins involved in LPS biosynthesis, antimicrobial resistance and phenazine production in *pmrB* mutants, and upregulation of proteins involved in adherence, lysozyme resistance and inhibition of the chloride ion channel CFTR, relative to wild-type strain LESB65. Accordingly, *pmrB* mutants are susceptible to antibiotic treatment but show enhanced adherence to airway epithelial cells, resistance to lysozyme treatment, and downregulate host CFTR expression. We propose that *P. aeruginosa pmrB* mutations in CF patients are subject to an evolutionary trade-off, leading to enhanced colonisation potential, CFTR inhibition, and resistance to host defences, but also to increased susceptibility to antibiotics.

## Abstract (web summary)

Mutations in gene *pmrB* are found in *Pseudomonas aeruginosa* isolates from cystic fibrosis patients. Here, Bricio-Moreno et al. show in a mouse model of respiratory infection that the mutations enhance bacterial adherence to epithelial cells and resistance to lysozyme, but also increase antibiotic susceptibility.

## Introduction

P*seudomonas aeruginosa* lung infections are the single biggest cause of mortality in people with cystic fibrosis (CF)^1,2^. Such infections are typically chronic, highly resistant to antimicrobial therapy and punctuated by periodic exacerbations. *P. aeruginosa* is ubiquitous in the environment and will thrive under diverse conditions^3^. Such adaptability is facilitated by a large and plastic genome^4^. However, we still know relatively little about the early adaptation events that take place within the host during infection.

Previous work by ourselves and others has suggested that the upper airways (the paranasal sinuses and nasopharynx) may function as a protected niche and “evolutionary nest” for *P. aeruginosa*, allowing prolonged colonisation in an environment with fewer immune defence agents and greater nutrient availability than the lung^5–8^. Gradual adaptation to the host environment paves the way for subsequent seeding into the lungs and establishment of chronic infection. When *P. aeruginosa* was cultured simultaneously from the sinuses and lungs of patients who had undergone Endoscopic Sinus Surgery, isolates from the two sites were genetically identical in 95% of patients^8^. Furthermore, *P. aeruginosa* form biofilms in the sinuses without triggering strong cellular inflammatory responses^8^.

We have previously used a chronic *P. aeruginosa* respiratory infection model in mice to demonstrate that, whilst host immune clearance mechanisms efficiently remove bacteria infecting the lung, colonisation persists in the nasopharynx for long periods^6^. The same study showed that prolonged colonisation is associated with subsequent reseeding events, whereby *P. aeruginosa* reappear in the lungs several weeks after initial immune clearance. Genome sequencing of isolates taken from nasopharynx and lungs one month post-infection revealed the presence of a single nucleotide polymorphism (SNP) resulting in a missense mutation in *pmrB*. This mutation was present in multiple isolates taken from different mice^6^. We have since compared the PmrB amino acid sequences of the Liverpool Epidemic Strain (LES)B65 used for the initial infection (DDBJ sequence read archive ERS2269679 (http://ddbj.nig.ac.jp/DRASearch/sample?acc=ERS2269679)) and PAO1 (GenBank AAG08163 (https://www.ncbi.nlm.nih.gov/protein/AAG08163.1)) and observed no differences, suggesting the SNP acquired by LESB65 in the mouse model was not a reversion mutation. An isolate with this *pmrB* SNP showed a greatly enhanced ability to colonise mouse lungs compared to both the LESB65 used in the original infection and an isolate taken from the mouse model that did not have a *pmrB* SNP (referred to as *pmrB* WT hereafter)^6^. Thus, our previous study suggested that acquisition of *pmrB* mutations early in infection appeared to confer a selective advantage in the establishment of lung infection. The Liverpool Epidemic Strain is a widely studied, transmissible lineage of *P. aeruginosa* from CF patients^9^. Isolates of this strain have been shown to have a modified LPS structure, lacking an O-antigen^9,10^, similar to other clinical isolates. Here, we describe the phenotypic changes associated with *pmrB* mutation that enable efficient lung colonisation.

Two-component regulatory systems are key regulators of virulence in bacteria, allowing the expression of multiple genes to be coupled to changes in the environment. *P. aeruginosa* has multiple two-component systems, amongst which PhoPQ and GacAS are probably the best characterised^11^. Phenotypic changes in *P. aeruginosa* that characterise the switch from acute to chronic infection are under the control of GacAS^12,13^. PmrAB is another such two-component system, involved in the modification of LPS in *P. aeruginosa*, and mutations in the *pmrAB* operon have been linked to resistance to cationic antimicrobial peptides^14^.

The mouse infection model from which we previously isolated *P. aeruginosa pmrB* SNP mutants did not include any antibiotic treatment^6^ and so we hypothesised that the selective pressure for mutation may come from host-derived antimicrobial peptides or that the phenotype conferred by the SNP mutation might be different from those previously reported. This was the starting point for the present study, wherein we describe a significantly altered phenotype of *P. aeruginosa* lacking *pmrB* or those carrying the in vivo acquired *pmrB* SNP and demonstrate that these changes aid in multiple facets of host colonisation by conferring resistance to host antimicrobials, enhanced adherence to host surfaces and an ability to modulate the local environment in a way that drives a CF lung phenotype. Our findings highlight the key role played by the *P. aeruginosa* PmrAB two-component regulatory system in regulating a wide range of biological processes and virulence mechanisms. Finally, we propose that the evolutionary trade-off associated with *pmrB* mutation may offer opportunities for therapeutic intervention, as enhanced in vivo fitness is offset by increased susceptibility to several classes of antibiotics.

## Results

### Loss-of-function *pmrB* mutations confer lysozyme resistance

**Fig. 1:**
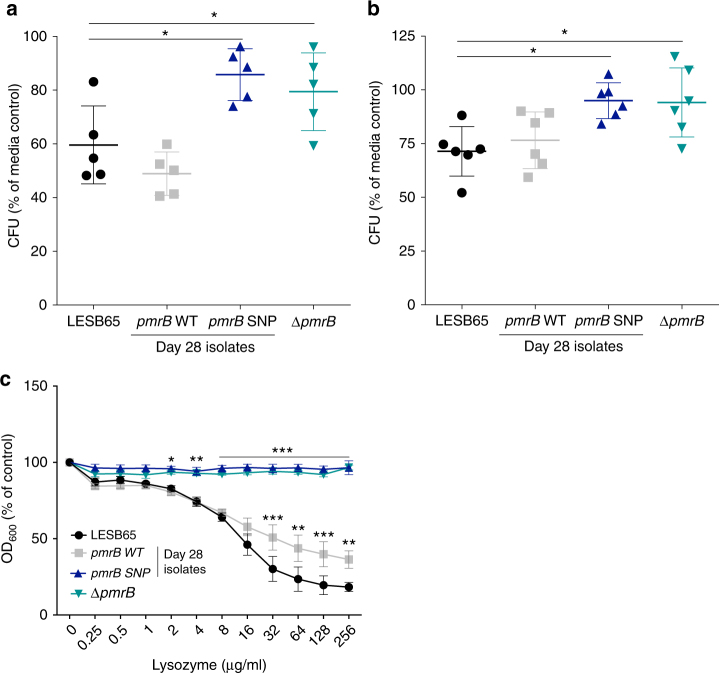
*P. aeruginosa pmrB* mutants are resistant to host defence molecules and lysozyme killing. Supernatants from phorbol 12-myristate 13-acetate (PMA)-stimulated (**a**) HL-60 or (**b**) mouse bone marrow neutrophil cultures were transferred to mid-log growth *P. aeruginosa*. Colony forming units (CFU) as a percentage of control. Control bacteria were exposed to supernatant from non-activated (no PMA) HL-60 cultures. **c** Optical density at 600 nm of *P. aeruginosa* grown in the presence of lysozyme (mean ± s.d.). Results are expressed as a percentage of the no-lysozyme control for each isolate and are a composite of 3 biological replicates per isolate, each containing 3 technical replicates. Asterisks (*) represent significant differences in ANOVA performed on CFU counts (**a**, **b**) or OD readings, with lysozyme as a covariant (**c**), with Dunnett’s multiple comparison test vs. LESB65. *p < 0.05, **p < 0.01, ***p < 0.005

Neutrophils are a major defence against bacterial infection of the respiratory tract and neutrophil degranulation releases antimicrobial peptides and enzymes^15^. In order to determine the effect of *pmrB* mutation on susceptibility to neutrophil killing, human HL-60 neutrophils and mouse neutrophils isolated from bone marrow were cultured with phorbol myrisate acetate (PMA) to induce degranulation and culture supernatants (containing granule contents) were transferred to LESB65, a *pmrB* deletion mutant on the LESB65 background (Δ*pmr*B) or two LESB65-derived isolates taken from 28 days post-infection in the mouse infection model, one with and one without a *pmrB* SNP (referred to as *pmrB* SNP and *pmrB* WT, respectively, hereafter, and as NP22_2 and NP22_4 in ref.^6^). A full description of the additional mutations in the LESB65-derived isolates, determined by PacBio sequencing, can be found in Supplementary Table 1. Isolates with mutated *pmrB* (*pmrB* deletion and SNP mutants) showed significantly enhanced resistance to killing by neutrophil-conditioned media (Fig. 1a, b). To determine whether resistance to a neutrophil-derived antimicrobial accounted for this phenotype, the four bacterial isolates were cultured with β-defensin 2, mCRAMP (the murine homologue of the human cathelicidin LL-37) or lysozyme. The two *pmrB* mutants showed significantly enhanced resistance to lysozyme killing (Fig. 1c), but not to β-defensin 2 or mCRAMP in two-way ANOVA analysis (Supplementary Fig. 1A and B). Lysozyme is abundant in the mammalian airway^16^ and is active against both Gram-positive and Gram-negative species^17,18^. Significant differences in resistance between isolates were observed at lysozyme concentrations within the physiological range found in saliva^19^. Lysozyme resistance obtained through *pmrB* mutation may, therefore, provide a selective advantage in the establishment of respiratory tract infection.

### Antimicrobial susceptibility in *pmrB* mutants

Mutations in the *pmrAB* system have previously been linked to changes in resistance to antibiotics^14,20^. To determine whether the phenotypic changes in *pmrB* mutants that conferred lysozyme resistance also influenced resistance to antibiotics, minimum inhibitory concentration (MIC) assays were performed (Table 1, Supplementary Fig. 2A-E). Strikingly, isolates with *pmrB* mutations were notably more susceptible to 6 of the 7 antibiotics tested, when antibiotic concentration required to inhibit 90% growth (MIC90) was determined (Table 1). These antibiotics included tobramycin and colistin, commonly used in the treatment of *P. aeruginosa* infections in people with CF. Similarly, *P. aeruginosa* with a transposon insertion in *pmrB* showed enhanced susceptibility to both tobramycin and colistin, relative to the parental PAO1 strain (Supplementary Table 2). Thus, on both the LES and PAO1 background, loss of *pmrB* function is associated with a relative increase in antibiotic susceptibility. The evolution of resistance to lysozyme at the cost of antibiotic susceptibility is an example of collateral sensitivity, as has been previously described for *P. aeruginosa pmrB* mutants, in which resistance to gentamicin was associated with penicillin sensitivity^21^.

**Table 1:** Antibiotic susceptibility of *Psuedomonas aeruginosa* isolates

Minimum inhibitory concentration (µg per ml) required for 50% or 90% growth inhibition. Median MIC50 and MIC90 from a minimum of 5 replicates (range 5–9) is in bold font, range is in brackets. The two/three most sensitive isolates for each antibiotic are shaded grey

Chronic *P. aeruginosa* lung infections are characterised by bacterial growth in biofilm that provides resistance to antibiotic treatment and host immune responses^22,23^. Although differences in tobramycin sensitivity between isolates were less pronounced when grown in an artificial sputum model (ASM) of biofilm growth, the increased susceptibility of *pmrB* mutants relative to LESB65 was evident at 4 and 8 µg/ml tobramycin (Supplementary Fig. 3A).

The effectiveness of lysozyme is also reduced in biofilms and in the chronically infected lung, due to electrostatic sequestration of the enzyme by infection-associated anionic biopolymers^24,25^. This was clear when LESB65 was grown in ASM, where it showed complete resistance to lysozyme at all concentrations tested (data not shown). However, when electrostatic sequestration by anionic biopolymers in ASM was negated through use of charge-engineered lysozyme^24,26^, the differential lysozyme resistance of *pmrB* mutants could still be observed (Supplementary Fig. 3B). This charge-engineering did not significantly enhance lysozyme killing of any of the four isolates when experiments were performed in planktonic culture (Supplementary Fig. 4).

### Proteomics analysis of *pmrB* mutants

**Fig. 2:**
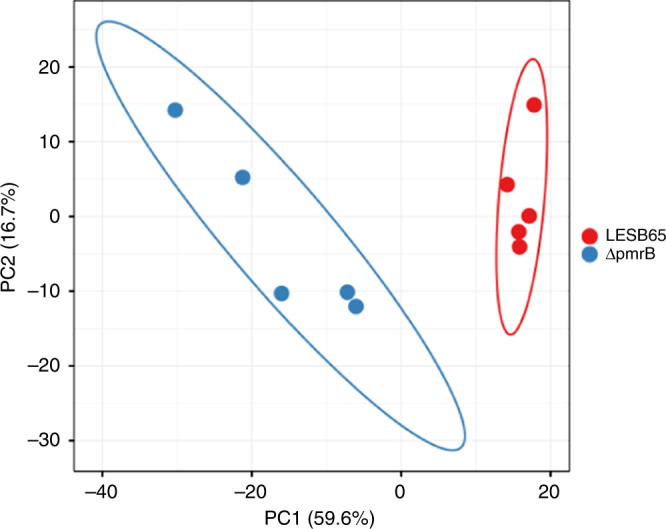
Principal component biplot of LESB65 and Δ*pmrB* proteomics data showing proteins with significantly different abundance between samples. Five biological replicates were performed for each isolate. Unit variance scaling is applied to rows; SVD with imputation is used to calculate principal components. *X* axis and *Y* axis show principal component 1 and principal component 2 that explain 59.6% and 16.7% of the total variance, respectively. Prediction ellipses are such that with probability 0.95, a new observation from the same group will fall inside the ellipse. PCA analysis of the full dataset, with all detected proteins, can be found in Supplementary Fig. 4

The dichotomous phenotypic changes in *pmrB* mutants, with enhanced resistance to a key host derived antimicrobial but increased susceptibility to antibiotics, suggested that *pmrB* mutation might lead to significant changes in bacterial gene expression, protein production or physiology. To explore this, we performed proteomic analysis of LESB65 and Δ*pmrB* grown in liquid culture. We found 216 proteins with significantly different abundance in LESB65 cultures compared to Δ*pmrB* (Fig. 2, Supplementary Data 1 and Supplementary Fig. 5), and these included the two proteins of the PmrAB two-component regulatory system, as well as key virulence factors (Table 2). Of the proteins identified as differentially abundant between the two isolates, 60% (129/216) were enriched in Δ*pmrB*.

**Table 2:** Differential protein abundance in LESB65 vs. Δ*pmrB* grown in liquid culture

Biological process	Protein name	Gene	Fold change	*q*-value
Type IV pili	Type IV fimbrial biogenesis protein PilV	*pilV* PA4551	6.14	0.003560888
	Type IV fimbrial biogenesis protein PilY2	*pilY2* PA4555	5.80	0.018144003
	Pilus associated adhesin PilY1	*pilY1* PA4554	2.33	0.026743646
CFTR inhibition	CFTR inhibitory factor CIF	*cif* PA2934	5.65	0.014178322
Lysozyme inhibition	Inhibitor of vertebrate lysozyme	*ivy* PA3902	2.66	0.049597254
Two-component signalling	PmrB: two-component signal sensor kinase	*pmrB* PA4777	−20.13	0.000337869
	PmrA: two-component signal response regulator	*pmrA* PA4776	−25.95	0.000615523
Phenazine biosynthesis	Phenazine biosynthesis protein PhzD	*phzD1 phzD2* PA1902 PA4213	−5.37	0.001208844
	Phenazine biosynthesis protein PhzE	*phzE1 phzE2* PA1903 PA4214	−3.93	0.001208844
	Pyridoxal 5’-phosphate synthase	*phzG1* PA4216	−2.98	0.01260054
	Phospho-2-dehydro-3-deoxyheptonate aldolase	*phzC1* phzC2 PA1901 PA4212	−6.89	0.000837624
	RND multidrug efflux membrane fusion protein MexE*	*mexE* PA2493	−5.39	0.029669765
	Anthranilate--CoA ligase	*pqsA* PA0996	−2.82	0.040457292
	BfmS	*bfmS* PA4102	−3.79	0.020754134
LPS modification	4-deoxy-4-formamido-L-arabinose-phosphoundecaprenol deformylase ArnD	*arnD* PA3555	−26.31	0.000200769
	Bifunctional polymyxin resistance protein ArnA	*arnA* PA3554	−18.63	0.000337869
	Undecaprenyl phosphate-alpha-4-amino-4-deoxy-L-arabinose arabinosyl transferase	*arnT* PA3556	−11.37	0.003786234
	UDP-4-amino-4-deoxy-L-arabinose--oxoglutarate aminotransferase	*arnB* PA3552	−7.17	0.01284628
	3-deoxy-D-manno-octulosonic-acid (KDO) transferase	*waaA* PA4988	−3.85	0.040439226
Antimicrobial resistance	CprA	*cprA* PA1559	−19.96	0.005901835
	CprA	*cprA* PA1560	−30.39	2.78965E-05
	Uncharacterised protein	PA4775	−22.89	0.00018425

Proteins dowregulated in Δ*pmrB* relative to LESB65 are denoted with a minus fold-change

*MexE is also involved in antibiotic resistance

Supportive of our phenotypic observations, several proteins with described roles in antimicrobial resistance were significantly more abundant in LESB65 than in Δ*pmrB*. These included the protein products of several genes of the *arn* locus, which is crucial to the synthesis of the lipid A component of bacterial lipopolysaccharide and which mediates resistance to cationic antimicrobials through addition of 4-amino-L-arabinose to lipid A^14^. Expression of the *arn* locus has been shown to be under the control of two-component systems including PhoPQ and PmrAB^14,27,28^. Using MALDI-TOF mass spectrometry, differences in lipid A traces were detected. Peaks corresponding to both penta-acyl lipid A and hexa-acyl lipid A were significantly reduced in Δ*pmrB* compared to the isogenic wild type LESB65 (Supplementary Fig. 6). Thus Δ*pmrB*, that demonstrated reduced expression of proteins of the *arn* locus, may also have reduced lipid A content.

Protein PA4775 (T224_RS56950 in LESB65), which was enriched in LESB65 but not Δ*pmrB* has also been linked to resistance to both antibiotics and oxidative damage via an LPS-dependent mechanism^29^. Other proteins with roles in antimicrobial resistance that were more abundant in LESB65 include CprA, which contributes to polymyxin resistance by an *arn*-independent pathway^30^, and the multidrug efflux pump protein MexE^31^.

Proteins involved in phenazine biosynthesis were also enriched in LESB65 as compared to Δ*pmrB*. Quantification of pyocyanin in cultures of LESB65, *pmrB* WT, *pmrB* SNP and Δ*pmrB*, confirmed that loss of PmrB function is associated with reduced pyocyanin production (Supplementary Fig. 7). Pyocyanin is a key virulence factor of *P. aeruginosa*, and it is unclear how its downregulation in Δ*pmrB* might contribute to increased survival in vivo. Notably however, pyocyanin is a stimulator of IL-8 release from the human airway epithelium and reduced production may aid evasion of neutrophil responses prior to establishment of biofilm^32,33^.

The lysozyme resistance observed with Δ*pmrB* is likely a consequence of increased production of the inhibitor of vertebrate lysozyme (Ivy) protein, enriched 2.66-fold in Δ*pmrB* relative to LESB65 (Table 2). An increase in cif expression was also detected in Δ*pmrB* and *pmrB* SNP using qPCR (Supplementary Table 3). Ivy is a proteinaceous inhibitor of lysozyme first described in *Escherichia coli*^34^. *E. coli ivy* knockout mutants exhibit growth defects in lysozyme-rich environments, including hen egg white and saliva^35^. However, Deckers et al. found no effect of *ivy* deletion in *P. aeruginosa* PAO1 on growth in lysozyme-containing saliva or egg white, perhaps due to compensatory lysozyme resistance mechanisms such as that mediated by MliC^36^.

### Modulation of CFTR by *pmrB* mutants

**Fig. 3:**
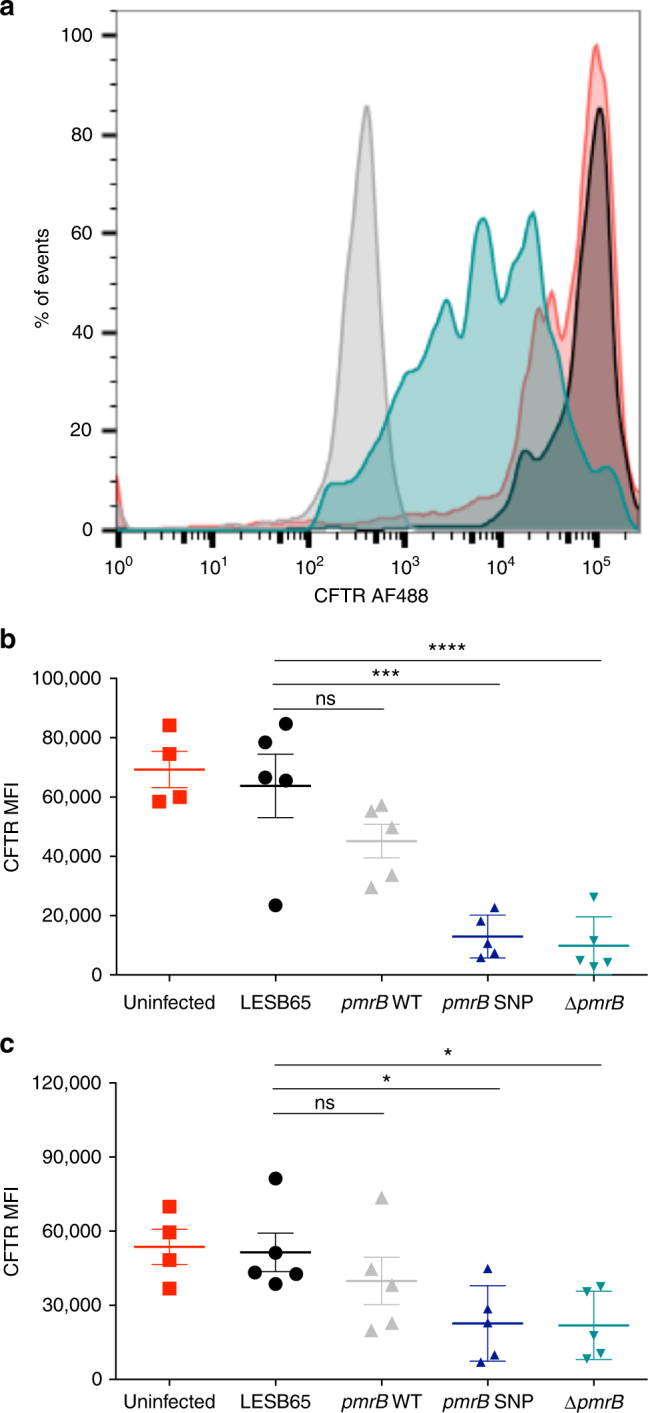
Δ*pmrB* downregulates CFTR expression on airway epithelial cells. **a** CFTR expression on uninfected (red), LESB65 infected (black) and Δ*pmrB* infected (blue) A549 airway epithelial cells as measured by flow cytometry and compared to isotype control staining (grey). CFTR alexa fluor 488 median fluorescent intensity (MFI) on stained (**b**) A549 and (**c**) Detroit cells. Data in (**b**) are a composite of two independent experiments with two-three biological replicates per condition in each experiment. Data in (**c**) are from a single experiment containing 4 biological replicates. Asterisks (*) represent significant differences in one-way ANOVA with Dunnett’s multiple comparison test. *p < 0.05, ***p < 0.001, ****p < 0.0001

The cystic fibrosis transmembrane conductance regulator (CFTR) inhibitory protein Cif was 5.65-fold more abundant in Δ*pmrB* than in LESB65 (Table 2). An increase in *cif* expression was also detected in Δ*pmrB* and *pmrB* SNP using qPCR (Supplementary Table 3). Cif inhibits endocytic recycling of CFTR in human epithelial cells^37^, decreasing apical membrane expression of the protein^38^. Furthermore, Cif is expressed in the CF lung^38^, suggesting that *P. aeruginosa* can phenocopy the CF lung environment, potentially facilitating colonisation and chronic infection. To determine whether the high levels of Cif measured in Δ*pmrB* samples was of functional relevance, LESB65 and Δ*pmrB* were cultured with A549 human pulmonary epithelial cells and incubated overnight before CFTR expression was assessed by flow cytometry. Strikingly, although A549 CFTR expression was unaffected by co-culture with LESB65, significantly decreased CFTR expression was evident on A549 cells cultured with Δ*pmrB* (Fig. 3a, b). Similarly, *pmrB* SNP, but not *pmrB* WT was able to reduce CFTR expression on A549 cells (Fig. 3b) and the same phenotypes were observed when Detroit human pharyngeal epithelial cells were used (Fig. 3c). This raises the possibility that the success of *pmrB* mutants in vivo may reflect an ability to modulate the host environment to create conditions conducive to bacterial growth.

### Loss of *cif* or *ivy* attenuates colonisation potential

**Fig. 4:**
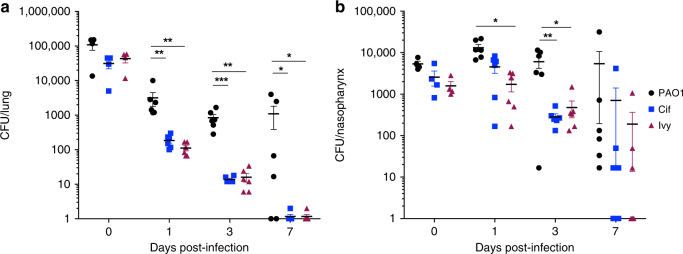
Cif and Ivy contribute to establishment of lung infection. Mice were infected by intranasal instillation of 1 × 10^6^ colony forming units (CFU) *P. aeruginosa* PAO1, or PAO1 with transposon insertions in *cif* or *ivy*. Mice were culled immediately after infection (*n* = 4 per group) or at 1, 3 and 7 days post-infection (*n* = 6 per group) and CFU in (**a**) lung and (**b**) nasopharynx determined by plating of tissue homogenates onto selective agar. Data are from a single experiment. *p < 0.05, **p < 0.01, ***p < 0.001 in two-way ANOVA with Dunnett’s multiple comparison test vs. PAO1

To determine whether Ivy and Cif make contributions to *P. aeruginosa* colonisation and survival in vivo, PAO1 transposon mutants with insertions in *ivy* or *cif* were compared to the parental PAO1 strain in a mouse inhalation infection model^6^. Lack of either *Cif* or *Ivy* led to a significant defect in establishment of infection that was more pronounced in lung than nasopharynx (Fig. 4a, b). *Cif* and *Ivy* mutant lung CFU were significantly reduced vs. PAO1 at days 1, 3 and 7 post-infection and infection was cleared from lung in 5/6 *Cif* and *Ivy*-infected mice, but only 2/6 PAO1-infected mice (Fig. 4a).

### Enhanced airway adhesion and motility in *pmrB* mutants

**Fig. 5:**
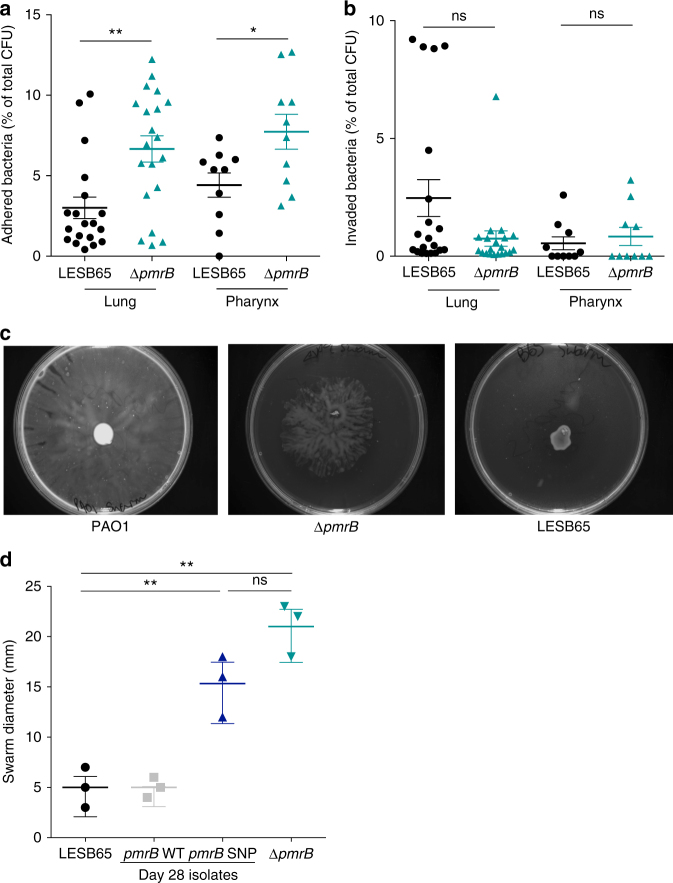
Pili-dependent processes in LESB65 and Δ*pmrB*. **a** Adhesion and (**b**) invasion of LESB65 and Δ*pmrB* to A549 human alveolar epithelial cells (lung) and human Detroit pharyngeal cells (pharynx). Results are expressed as a percentage of total bacterial numbers per well. Data are a composite of four (lung) or two (pharynx) independent experiments, each containing 5 replicate wells per experimental group. **c** Swarming motility of PAO1, Δ*pmrB*, and LESB65 on semisolid agar. (**d**) Swarm diameter of isolates grown on semisolid agar. Data points shown are from three independent experiments. *p < 0.05, **p < 0.01 and ns non-significant in unpaired *t*-test with Welch’s correction (**a, b**) or one-way ANOVA with Tukey post-test (**d**)

Several proteins involved in type IV pili biosynthesis were significantly more abundant in Δ*pmrB* than in LESB65. Type IV pili play crucial roles in mediating adhesion to host surfaces and in bacterial motility, but also in environmental sensing and virulence^39^. To determine whether increased abundance of type IV pili proteins in Δ*pmrB* enabled more efficient adherence to airway epithelial cells, adhesion/invasion assays were performed with A549 human alveolar epithelial cells and Detroit human pharyngeal epithelial cells (Fig. 5a, b). In all samples tested, less than 15% of bacteria were found to have adhered to epithelial cells after 2 h of co-culture, but the proportion of adhered bacteria was significantly higher in Δ*pmrB*-epithelial cell co-cultures than in LESB65-epithelial cell cultures (Fig. 5a). Adherence is key to persistence and establishment of biofilms^40^, and increased adherence in *pmrB* mutants may contribute to in vivo virulence.

The proportion of invaded bacteria found to be residing within A549 epithelial cells following triton X-induced lysis was higher for LESB65 than for Δ*pmrB* (Fig. 5b, *p* = 0.07 in unpaired *t*-test with Welch’s correction when considered as a percentage of total CFU, *p* < 0.01 when considered as a percentage of adhered CFU only). This may be a consequence of the CFTR downregulation induced by Δ*pmrB*, as CFTR has been implicated in *P. aeruginosa* cellular invasion^41–43^. No difference between LESB65 and ΔpmrB was observed for invasion of Detroit pharyngeal epithelial cells (Fig. 5b) that express lower levels of CFTR (Fig. 3c).

Motility in *P. aeruginosa* is mediated via both type IV pili and flagella^44^. Several pili proteins were more abundant in Δ*pmrB* than in LESB65 in the proteomic analysis. In motility assays, both Δ*pmrB* and *pmrB* SNP showed enhanced flagella-dependent and type IV pili-dependent swarming^45^ relative to LESB65 or *pmrB* WT (Fig. 5c, d). No difference in flagella-dependent swimming or pili-dependent twitching was observed (Supplementary Fig. 8A–D). Swarming takes place on the sort of viscous or semisolid surfaces typified by the mucous of the CF lung and swarming motility is thought to be linked to the expression of virulence factors^46^. Thus, enhanced swarming in Δ*pmrB* may be an adaptation to the host environment.

### Proteomics comparison of *pmrB* SNP with *pmrB* deletion mutant

**Fig. 6:**
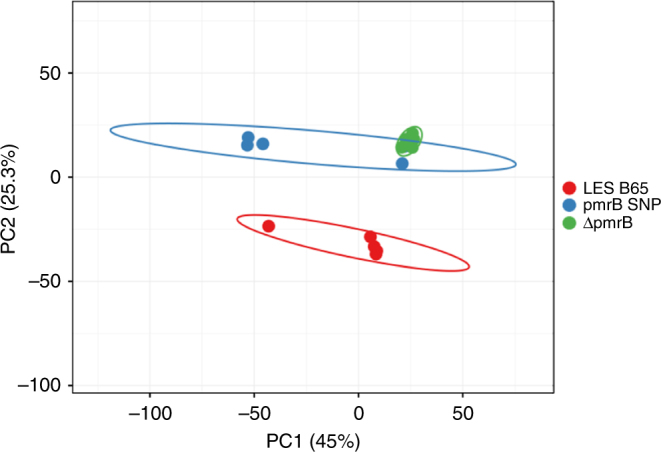
Principal component biplot of LESB65, *pmrB* SNP and Δ*pmrB*. Unit variance scaling is applied to rows; SVD with imputation is used to calculate principal components. *X* axis and *Y* axis show principal component 1 and principal component 2 that explain 45% and 25.3% of the total variance, respectively. Prediction ellipses are such that with probability 0.95, a new observation from the same group will fall inside the ellipse. *N* = 15 data points

Our in vitro data suggested that deletion of *pmrB* reproduced the phenotype of the mouse model *pmrB* SNP isolate. Direct proteomic comparison of LESB65, *pmrB* SNP and Δ*pmrB* confirmed this, with the two *pmrB* mutant strains clustering together, and away from LESB65 in principal component analysis (Fig. 6). PmrA (*p* = 2.76 × 10^−5^) and PmrB (*p* = 5.74 × 10^−10^) were significantly less abundant in *pmrB* SNP relative to LESB65, as proteins were involved in phenazine biosynthesis (phzG1, phzM, phzB1, phzA2), LPS modification (arnA, arnB, arnD, arnT, waaG) and antimicrobial resistance (CprA, PA4775). Type IV pili proteins were enriched in *pmrB* SNP vs. LESB65 (PilY1, PilF) (Supplementary Data 2). Peptide counts for Cif and Ivy were below the cut-off threshold for analysis in these experiments, but expression differences were confirmed by qPCR (Supplementary Table 3).

### Mutations in *pmrB* in clinical *P. aeruginosa* isolates

**Fig. 7:**
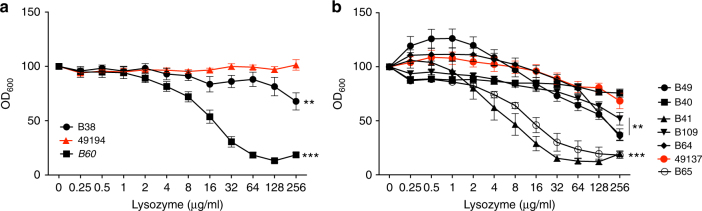
*pmrB* mutations are found in patient isolates and are associated with lysozyme resistance. Three isolates from one patient (**a**) and seven from another (**b**) were compared for lysozyme resistance. Isolates with at least one *pmrB* mutation are in red (49194 = R202H, 49137 = I73L, E82K, E225Q). (**a**, **b**) Growth in the presence of lysozyme expressed as a percentage of growth in LB (OD_600_). Data are presented as mean ± s.d. and are a composite of three independent experiments with each isolate run in triplicate. **p < 0.01, ***p < 0.005, ns non-significant, vs. 49194 (**a**) or 49137 (**b**) in ANOVA analysis performed on OD_600_ with lysozyme as a covariant

Although loss of *pmrB* appears to confer phenotypic changes that facilitate establishment of infection in vivo (Figs. 1, 3, 4, 5), it is also associated with increased susceptibility to antibiotics (Supplementary Fig. 3, Table 1). It is not clear, therefore, whether such mutations would be advantageous in clinical *P. aeruginosa* infection, where intensive antibiotic therapy is the norm. Sequencing of *pmrB* of 13 Liverpool Epidemic Strain (LES) isolates taken from four CF patients attending the Children’s CF centre in Liverpool between 1995 and 2004^47^ revealed the presence of SNPs in two isolates from two different individuals. A SNP in isolate 49,194 resulted in conversion of PmrB arginine 202 to histidine, whilst three SNPs in isolate 49,137 from a different patient led to isoleucine 73 to leucine, glutamate 82 to lysine, and glutamate 225 to glutamine conversions. Both isolates had amino acid changes (at position 202 in 49,194 and 225 in 49,137) in proximity to the conserved H-box region of PmrB. The *pmrB* SNP isolate from the mouse model that was used in this study and described previously also had an amino acid change in the H-box region (L256Q)^6^. Clinical isolates 49,194 and 49,137 both showed high levels of resistance to lysozyme and were amongst the most susceptible to antibiotics of the isolates from each of the two individuals (Fig. 7 and Supplementary Table 4). Furthermore, both 49,194 and 49,137 showed evidence of increased *cif* and *ivy* expression relative to paired isolates from the same patients (Supplementary Table 5). Considerable variation in both lysozyme and antibiotic resistance was observed amongst isolates from a single patient, highlighting the within-patient phenotypic diversity of *P. aeruginosa* (Fig. 7 and Supplementary Table 4). Thus, *pmrB* mutations are evident in clinical *P. aeruginosa* isolates and are associated with similar phenotypic traits to those identified in the isolates from our mouse model.

## Discussion

**Fig. 8:**
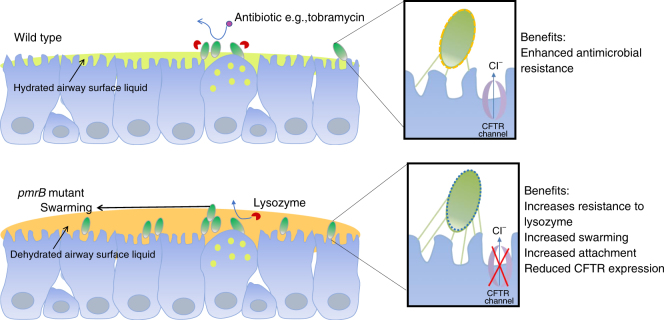
Proposed mechanism for enhanced infection potential of *pmrB* mutants. Protein expression changes associated with *pmrB* mutation may confer an ability to reproduce the phenotypic features of the CF lung via inhibition of host CFTR. Increased expression of type IV pili aids colonisation and adhesion to airway epithelium. Resistance to lysozyme associated with increased ivy production enables persistence in the face of immune pressure. The airway surface liquid is depicted in green for wild type and yellow for the *pmrB* mutant to denote dehydration resulting from CFTR inhibition

Based on our observations, we propose a model whereby *pmrB* mutants are selected during early infection due to the advantages they confer in colonisation of the host niche (summarised in Fig. 8). Furthermore, as part of a genotypically and phenotypically diverse *P. aeruginosa* population, such as exists in the CF lung^48–53^, *pmrB* mutation may confer a population-wide benefit through modulation of the host environment via CFTR inhibition. Given the evolutionary trade-offs associated with *pmrB* mutation, including enhanced susceptibility to antibiotics, it may be that the maintenance of population diversity is key during clinical infections.

Many of our observations have been made using isolates adapted to the murine lung, which serves as a useful model for human lung infections but does not fully reproduce the features of the CF lung environment. We chose isolate LESB65 for these studies due to its ability to establish long-term infection of the murine respiratory tract^6^. However, as a clinical CF isolate, it might be argued that it is already adapted to the environment of the respiratory tract. However, *P. aeruginosa* infection of the CF is characterised by genotypic and phenotypic diversity^48–53^, and no single isolate is likely to capture the extent of this diversity. Different mutations are likely to provide fitness benefits at different stages of infection, and *pmrB* mutation appears most important during early establishment of colonisation. In established infection, other genotypes may take over or coexist in a diverse population. We have demonstrated that *pmrB* mutations occur amongst clinical isolates (Fig. 8)^54^ and that they have the capacity to make significant contributions to success of infection at the population level. Analysis of a larger pool of clinical isolates from the early stages of infection, to quantify the prevalence of loss-of-function *pmrB* mutations, and the impact on antimicrobial susceptibility, would provide further insight into this crucial period. Such studies will help determine the extent to which the phenotypes of lysozyme resistance and environmental modulation, we identify here are selected during infection in people with CF.

Collectively, these data highlight the range of *P. aeruginosa* virulence and regulatory mechanisms linked to the PmrAB two-component regulatory system. Mutations in *pmrB* may be a sentinel early event in adaptation to the host respiratory tract. The evolutionary trade-off associated with *pmrB* mutation, whereby increased in vivo fitness is partially offset by antibiotic susceptibility, may offer opportunity for intervention with intensive eradication therapy in early infection. Such an approach would rely on early detection of infection and our earlier findings that the upper respiratory tract acts as an evolutionary nest for *P. aeruginosa* suggests that nasal swabbing could offer a route to earlier diagnosis and treatment in high risk individuals, including those with CF.

## Methods

### Bacterial isolates and gene deletion mutant isolates

LESB65 WT, *pmrB* WT and *pmrB* SNP were used and described in a previous study^6^. The LESB65 ∆*pmrB* mutant was created specifically for this study. Upstream and downstream regions of *pmrB* were amplified using Q5 high fidelity DNA polymerase (NEB) using primer sets PmrB1_Xbal 5′-TACTATTCTAGAGGACGACCTGCTGCTCG-3′/PmrB2_Kpn1 5′AATCCAGGTACCCGGGGGACTCCGGTAGGC-3′–and PmrB3_Kpn1 5′CCAAATGGTACCTAGCCTGCGCATTGGCCGGG-3′/PmrB4_EcoRI 5′CCTTGTGAATTCCGGCGAACGCCTGGAGCTA-3′. Restriction sites are underlined. Following digestion with Xbal and Knp1 (upstream regions) or Knp1 and EcoRI (downstream region), the amplicons were ligated into the Xbal/EcoRI sites of pEX18Gm “donor” and transformed into *Escherichia coli* GT115 (selected on gentamicin 10 mg per L). LESB65 WT and a helper *E. coli* GT115 (pRK2013) were grown on nutrient agar containing 25 mg/L trimethoprim and 25 mg per L kanamycin respectively. *P. aeruginosa* LESB65 was grown at 42 °C and *E. coli* at 30 °C, overnight. 1 ml of overnight culture was centrifuged at 6500 r.p.m. and resuspended gently in SOB. Recipient, donor, and helper were mixed at a ratio of 3:1:1 ratio and 150 µl were pipetted onto the surface of a SOB plate. These were incubated for 20 h at 30 °C. Bacteria were removed from the plate and reinoculated onto agar plates containing 25 mg per L trimethoprim and 200 mg per L gentamicin. Colonies were grown overnight and then negatively selected based on growth on 5% 9 (v/v) LB Sucrose (with trimethoprim and gentamicin) plates. Colonies were then grown in LB with trimethoprim (25 mg per L) and then plated to assess for no gentamicin sensitivity. Knockout mutants were confirmed by Sanger sequencing of the *pmrB* region (Source Biosciences). PAO1 and transposon mutants from the Two-Allele library were purchased from the Manoil lab (University of Washington) and transposon insertion/gene absence confirmed by PCR (data not shown). Cif and Ivy mutant references are Cif-G08::ISlacZ/hah and PA3902-F10::ISphoA/hah.

### Neutrophil degranulation resistance assays

Mouse bone marrow macrophages were isolated from mouse femurs and tibias as previously described^55^. Briefly, a 25-gauge needle was used to flush cells from bones and filtered through a 100 µm cell strainer. After washing in cold PBS, cells were separated by histopague 1119/1077 density gradient centrifugation. Purity was confirmed by flow cytometry staining for Ly6G and CD11b. HL-60 neutrophils (5 × 10^5^ per ml) or mouse bone marrow macrophages (5 × 10^5^ per ml) were cultured with phorbol myrisate acetate (PMA) for 4 h to induce degranulation and cell-free culture supernatants were transferred to 1 × 10^5^ pelleted mid-log LESB65, *pmrB* WT, *pmrB* SNP and Δ*pmrB*. CFU were determined after 1 h exposure, by serial dilution onto LB agar.

### Minimum inhibitory concentration

A broth microdilution method was used to determine the MIC of various host antimicrobial peptides and antibiotics on the *P. aeruginosa* strains LESB65, *pmrB* WT, *pmrB* SNP and Δ*pmrB*. Following bacterial growth on LB agar plates at 37 °C for 18 h, a single colony was inoculated into 5 ml of LB broth and incubated for a further 18 h at 37 °C and 180 r.p.m. A fresh dilution in LB broth was made by incubating 200 µl of the overnight culture in 5 ml of fresh LB media. A hundred microliters from the fresh bacterial culture were incubated in 96 well-plates with 100 µl of 1:2 serially diluted antimicrobial peptide in LB. After a 24 h static incubation at 37 °C the OD_600_ was determined to assess bacterial growth. The antimicrobial peptides used were the cathelicidin antimicrobial peptide LL37, lysozyme from chicken egg white (Sigma-Aldrich, Gillingham, UK), and β-defensin. The concentration range of the antimicrobial peptide used in the MIC assays varied between peptides: 0-100 µg per ml for LL37, 0-10 µg per ml for β-defensin and 0-250 µg per ml for lysozyme.

### MIC assay in artificial sputum media

The artificial sputum media was prepared as previously described^56,57^ using DNA from fish sperm (Sigma-Aldrich), mucin from porcine stomach (Sigma-Aldrich), the iron-chelator diethylene triamine pentaacetic acid (Sigma-Aldrich), NaCl (Sigma-Aldrich), KCl (Sigma-Aldrich), egg yolk emulsion (Sigma-Aldrich) and all essential and non-essential amino acids (Fisher Scientific, Loughborough, UK and Sigma-Aldrich). Similarly to the MIC assay in LB, the bacterial isolates LESB65, *pmrB* WT, *pmrB* SNP and ∆*pmrB* were grown in LB agar plates at 37 °C for 18 h. Following incubation, a single colony was inoculated into 5 ml of LB and incubated for 18 h at 37 °C and 180 r.p.m. The overnight culture was diluted into fresh LB broth to an OD_600_ of 0.05. A 1:100 dilution of the bacterial solution was done in sterile ASM and 1.8 ml of the solution was incubated with 200 µl of engineered lysozyme^25^ or tobramycin (Flynn Pharma LTD, Hertfordshire, UK) at different concentrations for 24 h at 37 °C and 50 r.p.m. Following incubation, the ASM biofilm was dissolved using Sputasol (Oxoid, Basingstoke, UK). The CFU from each biofilm was determined using the Miles and Misra technique^58^.

### Sequencing analysis

Three *P. aeruginosa* LESB65 isolates were sequenced using two Pacific Biosciences RS-II SMRT-cells per isolate (a total of six SMRT-cells) using P2/C4 chemistry: The parental isolate (LESB65), *pmrB* SNP, and *pmrB* WT. Subreads were assembled using canu v.1.7.11, and single nucleotide polymorphisms were extracted and annotated using SNIPPY, which takes fake reads generated from the canu consensus sequence, maps these to a reference sequence (*P. aeruginosa* LESB58) using bwa-mem2 and extracts SNP using Freebayes3 and SNPEff4. As Pacific Biosciences sequencing is prone to systematic insertion/deletion errors in homopolymeric tracts, these were removed from the subsequent analysis. Data are available under accessions ERS2269679 (http://ddbj.nig.ac.jp/DRASearch/sample?acc=ERS2269679) (Parent); ERS2269680 (http://ddbj.nig.ac.jp/DRASearch/sample?acc=ERS2269680) (*pmrB* SNP) and ERS2269681 (http://ddbj.nig.ac.jp/DRASearch/sample?acc=ERS2269681) (*pmrB* WT).

### Proteomic analysis

The LESB65, *pmrB* SNP and ∆*pmrB* strains were incubated on LB agar plates for 18 h at 37 °C. A sweep of colonies were inoculated into 5 ml of LB broth and incubated for a further 18 h at 37 °C and 180 r.p.m. A fresh dilution in LB broth was made by incubating 1.2 ml of the overnight culture into 28.8 ml of fresh LB media. The bacteria were incubated at 37 °C and 180 r.p.m. to an OD_600_ between 0.5 and 0.6 and pelleted down at 4500 r.p.m. for 12 min. The pellet was washed once in phosphate buffered saline and stored at −80 °C until processing for proteomic analysis. Five replicates per condition were used. Bacterial cell pellets were lysed by sonication 1% (w/v) SDC in 50 mM ammonium bicarbonate. Samples were heated at 80 °C for 15 min before centrifugation at 12,000×*g* to pellet debris. The supernatant was retained, and proteins reduced with 3 mM DTT (Sigma) at 60 °C for 10 min, cooled, then alkylated with 9 mM iodoacetamide (Sigma) at RT for 30 min in the dark; all steps were performed with intermittent vortex-mixing. Proteomic-grade trypsin (Sigma) was added at a protein:trypsin ratio of 50:1 and incubated at 37 °C overnight. SDC was removed by adding TFA to a final concentration of 0.5% (v/v). Peptide samples were centrifuged at 12,000×*g* for 30 min to remove precipitated SDC.

### NanoLC MS ESI MS/MS analysis

Peptides were analysed by on-line nanoflow LC using the Ultimate 3000 nano system (Dionex/Thermo Fisher Scientific). Samples were loaded onto a trap column (Acclaim PepMap 100, 2 cm × 75 μm inner diameter, C18, 3 μm, 100 Å) at 5 μl min−1 with an aqueous solution containing 0.1% (v/v) TFA and 2% (v/v) acetonitrile. After 3 min, the trap column was set in-line an analytical column (Easy-Spray PepMap® RSLC 50 cm × 75 μm inner diameter, C18, 2 μm, 100 Å) fused to a silica nano-electrospray emitter (Dionex). The column was operated at a constant temperature of 35 °C and the LC system coupled to a Q-Exactive HF mass spectrometer (Thermo Fisher Scientific). Chromatography was performed with a buffer system consisting of 0.1% formic acid (buffer A) and 80% acetonitrile in 0.1% formic acid (buffer B). The peptides were separated by a linear gradient of 3.8–50% buffer B over 90 min at a flow rate of 300 nl per min. The Q-Exactive HF was operated in data-dependent mode with survey scans acquired at a resolution of 60,000. Up to the top 10 most abundant isotope patterns with charge states +2 to +5 from the survey scan were selected with an isolation window of 2.0Th and fragmented by higher energy collisional dissociation with normalised collision energies of 30. The maximum ion injection times for the survey scan and the MS/MS scans were 100 and 45 ms, respectively, and the ion target value was set to 3E6 for survey scans and 1E5 for the MS/MS scans. MS/MS events were acquired at a resolution of 30,000. Repetitive sequencing of peptides was minimised through dynamic exclusion of the sequenced peptides for 20 s.

### Protein identification and quantification

Thermo RAW files were imported into Progenesis QI for proteomics (version 4.1, Nonlinear Dynamics). Runs were time aligned using default settings and using an auto selected run as reference. Peaks were picked by the software using default settings and filtered to include only peaks with a charge state between +2 and +7. Spectral data were converted into.mgf files with Progenesis QI for proteomics and exported for peptide identification using the Mascot (version 2.3.02, Matrix Science) search engine. Tandem MS data were searched against translated ORFs from *Pseudomonas aeruginosa* strain PAO1 (Uniprot reference proteome, UP000002438, December 2016) and a contaminant database (cRAP, GPMDB, 2012) (combined 5733 sequences; 1,909,703 residues). The search parameters were as follows: precursor mass tolerance was set to 10 p.p.m. and fragment mass tolerance was set as 0.05 Da. Two missed tryptic cleavages were permitted. Carbamidomethylation (cysteine) was set as a fixed modification and oxidation (methionine) set as variable modification. Mascot search results were further validated using the machine learning algorithm Percolator embedded within Mascot. The Mascot decoy database function was utilised and the false discovery rate was <1%, while individual percolator ion scores >13 indicated identity or extensive homology (*p* < 0.05). Mascot search results were imported into Progenesis QI for proteomics as XML files. Peptide intensities were normalised against the reference run by Progenesis QI for proteomics and these intensities were used to highlight relative differences in protein expression between sample groups. Only proteins with 2 or more identified peptides were included in the dataset. Statistical analysis (ANOVA) of the data was performed using Progenesis QI for proteomics to identify significantly (*p* < 0.05, *q* ≤ 0.05, relative fold change ≥2) differentially expressed proteins. PCA plots were created using ClustVis using proteins with 2 or greater identified peptides, and *q* < 0.05. Unit variance scaling was applied to rows; SVD with imputation was used to calculate principal components.

### MALDI-MS of Lipid A modifications

Cultures were initially prepared as described for the proteomic analysis. Bacteria were aliquoted (1 ml) into a 1.5 ml microtube prior to heat-inactivation for 1 h at 90 °C. The heat-inactivated bacteria were washed three times with 0.5 ml of double distilled water and centrifuged at 9000×*g* for 5 min and suspended in double distilled water at a final concentration of about 10^4^ to 10^5^ bacteria per μl. Four biological replicates (each with 4 technical replicates) were performed on the LESB65 WT and the ∆pmrB knockout mutant. Suspensions of 10^4^ to 10^5^ bacteria per µL were stored at −80 °C until required. 0.5 µL of bacterial suspension was spotted onto a stainless steel MALDI plate, 0.5 µL of the matrix, 2,5-dihydrobenzoic acid (DHB) made to a final concertation of 10 mg/mL in a 90:10 chloroform/methanol solution, was spotted on top of the bacterial suspension. MALDI q-TOF analysis were acquired using a Synapt G2-Si (Waters, UK). Negative ion data were recorded in resolution mode, with a mass range of *m*/*z* 1000–2200. Data was acquired for 180 s per sample with a scan rate of 1 scan per second and a laser repetition rate of 500 Hz. Average Spectra per acquisition were produced using MassLynx V4.1 (Waters, UK) and processed using the automatic peak detection with background subtraction. Peak intensities were normalised to the average total ion current of the acquisition. A peak was detected at *m*/*z* meas 1615.9873 corresponds to hexa-acyl Lipid A (*m*/*z* calc 1615.9879, Δppm −0.37). Another peak was detected at *m*/*z* meas 1445.8531 corresponds to penta-acyl Lipid A (*m*/*z* calc 1445.8572, Δppm −2.8).

### Pyocyanin production

Pyocyanin assays were carried out on culture supernatants during growth in LB as described previously^47^. The amount of pyocyanin in culture supernatants was quantified by measuring the OD_695_ value. For overnight cultures, it was important to shake the culture vigorously before obtaining the supernatant. This is to ensure that all of the pyocyanin present is in the oxidised form. We used a simple broth test to identify strains with increased blue/green pigment production compared to strain PAO1. Following overnight growth in universals containing 5 ml Luria broth, with shaking at 200 r.p.m. Full pyocyanin production assays were carried out using 250 ml flasks containing 50 ml LB and absorbance at 695 nm for supernatants and 600 nm for cell cultures.

### CFTR expression in airway epithelial cells

A549 alveolar epithelial cells (ATCC CCL-185) and Detroit 562 pharyngeal cells (ATCC CCL-138) were obtained from the American Type Culture Collection (Manassas, USA) and epithelial phenotype confirmed by microscopy and flow cytometry. Cells were confirmed mycoplasma-free before performing experiments. Airway cells, at 10^5^ per well, were seeded in 12 well plates and grown until the monolayer of cells were confluent, then infected with bacteria (CFU 10^4^) and incubated overnight at 37 °C. Cells were washed once with PBS removed with a cell scraper before four wells from each treatment group were pooled and CFU counts performed. A549 cells were washed and then incubated with anti-CD16/32 (Human TruStain FcX, BioLegend, cat. 422301) in PBS 1% (w/v) BSA for 30 min at room temperature. Cells were washed and then fixed and permeabilised (BD Biosciences) before staining with anti-CFTR AF488 (AbCam, cat. ab2784) in permeabilisation buffer (BD Bioscience) for 30 min at room temperature. Flow cytometry was performed using the BD FACSCelesta (BioSciences, Warrington, United Kingdom) and data analysed using Flow Jo v8.3 (Tree Star Inc, USA). Flow cytometry experiments were performed twice with two-three biological replicates per assay.

### Mouse infections

All mouse infection work was performed at the University of Liverpool with prior approval by the UK Home Office and the University of Liverpool Ethics Committee. The female BALB/c mice of 6-8 week old (Charles River, UK) were used for infection experiments and housed in individually ventilated cages for one week to acclimatise prior to infection. Sample size determination was performed as previously described, informed from previous work comparing *P. aeruginosa* infection density in mice^6^. Mice were randomly assigned to a cage (experimental group) on arrival at the unit by staff with no role in study design. 2 × 10^6^ colony forming units of PAO1 or PAO1 transposon mutants were instilled into the nares of mice that had been lightly anaesthetised with a mixture of isoflurane and oxygen. Cage labels were reversed to ensure researchers were blinded to experimental groups. Mice were monitored for signs of disease and culled at pre-determined time points. Nasopharyngeal tissue and lungs were removed post-mortem and homogenised in 3 ml PBS using an IKA T10 handheld tissue homogeniser (IKA, USA), before serial dilution onto *Pseudomonas* selective agar (Oxoid, UK) for enumeration of infectious burden. Following enumeration, researchers were unblinded. No animals were excluded from analysis.

### Adhesion and invasion of airway epithelial cells

Differential adhesion and invasion assay performed as previously described^59,60^. Briefly, A549 alveolar epithelial cells (ATCC CCL-185) or Detroit 562 pharyngeal cells (ATCC CCL-138) (both at 10^5^ per well) were seeded in 2 × 24 well plates and grown until confluent then infected with bacteria (CFU 10^4^) and incubated for two hours. Cells were washed five times with PBS to remove non-adherent bacteria and for invasion assay, samples were incubated for a further two hours in media with 600 mg per ml gentamicin to kill extracellular and adherent bacteria (adhesion control was performed without gentamicin). The cells were washed three times with PBS and 100 µl trypsin added to remove cells from the plate (incubated for 10 min), then cells lysed with 100 µl 0.05% (v/v) Triton x100 in incubator for 10 min. Samples were plated on LB agar in 10-fold dilutions up to 10^−6^ using the Miles & Misra technique^58^ and incubated at 37 °C for 48 h, at which point bacterial CFU were counted. Experimental conditions were performed in duplicate and repeated five times. Adhesion CFU per ml was calculated by removing invasion counts from invasion plus adhesion counts.

### Bacterial motility assays

Swim and swarm petri dish plates were inoculated on the surface as described previously^61,62^, then incubated for 14 and 16 h, respectively, at 37 °C. Swim plates were first loosely wrapped in cling film to prevent dehydration, and incubated without inversion. The visible diameter of bacterial growth was measured at the widest point after incubation. Twitching motility was determined by subsurface stab assay of a colony of bacteria deep into LB agar until it touched the agar-dish interface^63^, then motility zone measured after incubation for 24 h at 37 °C. To visualise the twitching motility diameter, agar was removed with tweezers and plates stained with 10 ml 0.25% (w/v) crystal violet for 30 min before rinsing with water. An isolate with diameter < 10 mm at widest point was considered twitching motility impaired, with experiments repeated five times.

### PCR amplification and sequencing of *pmrB*

PCR amplification was carried out as described previously^64^. Amplification of the *pmrB* region was carried out using the oligonucleotide primer sets pmrBF (5′- CCTACCCCTCTCGCTGAAG-3′)/pmrBR (5′-GATGTTCATCCGGGTCTCCT-3′). PCR amplicons were purified using Qiaquick PCR purification columns (Qiagen) and sequenced by Source Bioscience using the same oligonucleotide primers employed in the PCR amplification.

### Quantitative real-time PCR (qPCR) and analysis

qPCR was performed using the Rotor-Gene Q cycler (Qiagen) as described previously^6^. Specific forward and reverse primers were used to target *cif* (cif_F-TGGCACTTCAGTTTCTTCGC and cif_R-TGTTGCTGGAATGGGACTTG primerS) and *ivy* (ivy_F-GAGTATCCAGACTGCTCTCCC and ivy_R-TGGGGTTTGCAACTGTTGG primers). Each qPCR was performed with three biological replicates and in duplicate on each run. A standard curve of known copy number was performed in each run. Obtained data were analysed using the Rotor-Gene Q series software (Qiagen). Relative expression levels of genes *cif* and *ivy* were calculated using the two-standard curve method. Genes *proC* and *rpoD*, encoding the pyrroline-5-carbocylate reductase and sigma factor RpoD, respectively, were used as reference genes. A standard curve was constructed for each target and reference genes.

### Statistical analysis

Statistical analysis was performed in GraphPad Prism V 6.0 (GraphPad Software, La Jolla, USA) and data were tested for normality and to define the variance of each group tested. All multi-parameter analyses included corrections for multiple comparisons and data are presented as mean ± standard deviation (SD) unless otherwise stated. For MIC determination, median values are stated and odd numbers of replicates performed to avoid derived values.

### Data availability

PacBio sequence data are available from DDBJ sequence read archive under accessions ERS2269679 (LESB65), ERS2269680 (*pmrB* SNP) and ERS2269681 (*pmrB* WT). The mass spectrometry proteomics data have been deposited to the ProteomeXchange Consortium via the PRIDE^65^ partner repository with the dataset identifiers PXD009705 and 10.6019/PXD009705. All other data supporting the findings of the study are available in this article and its Supplementary Information files, or from the corresponding authors on request.

## Electronic supplementary material

Supplementary Information

Description of Additional Supplementary Information

Supplementary Data 1

Supplementary Data 2

Supplementary Data 3

Supplementary Data 4
