# Supplementary material for: Evolutionary trade-offs associated with loss of PmrB function in host-adapted Pseudomonas aeruginosa
Source: Nat Commun. 2018 Jul 6;9:2635. doi: 10.1038/s41467-018-04996-x (PMC6035264; doi:10.1038/s41467-018-04996-x)
Supplement: Supplementary file 1 — Supplementary Information [file 41467_2018_4996_MOESM1_ESM.pdf]

Evolutionary trade-offs associated with loss of PmrB function in host-adapted *Pseudomonas aeruginosa*

Bricio-Moreno *et al.*

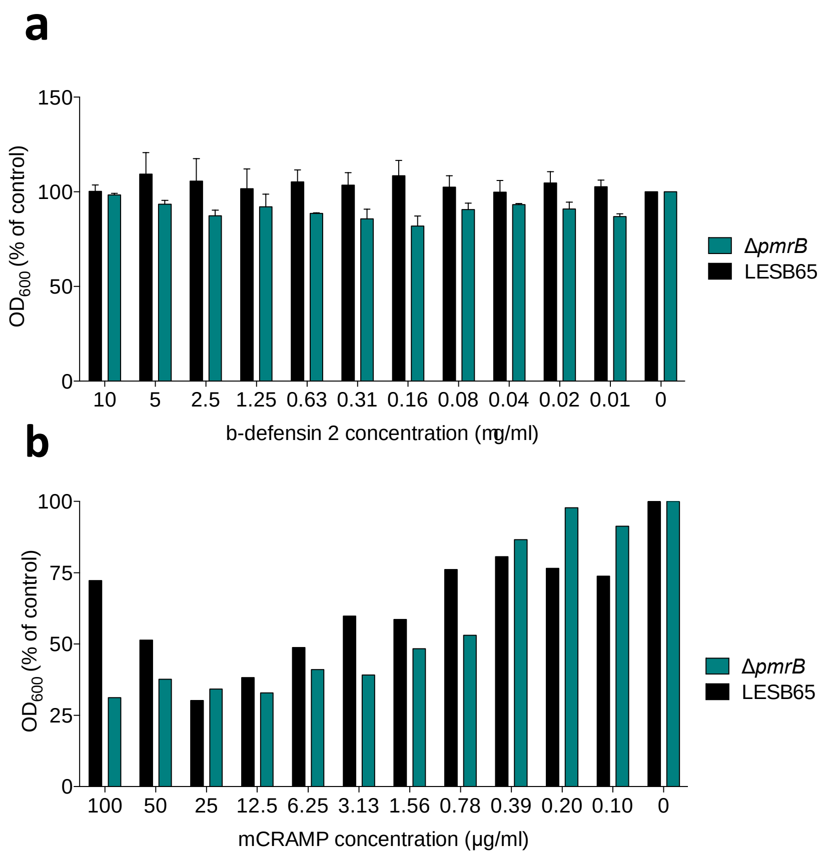

Supplementary Figure 1. LESB65 and Δ*pmrB* show comparable resistance to the host-derived antimicrobials β-defensin 2 and m-CRAMP. Bacteria were cultured overnight in LB in the presence of **(a)** β-defensin 2 or **(b)** mCRAMP. Data are from a single experiment and presented as OD_{600} as a percent of growth in LB alone (mean ±SD).

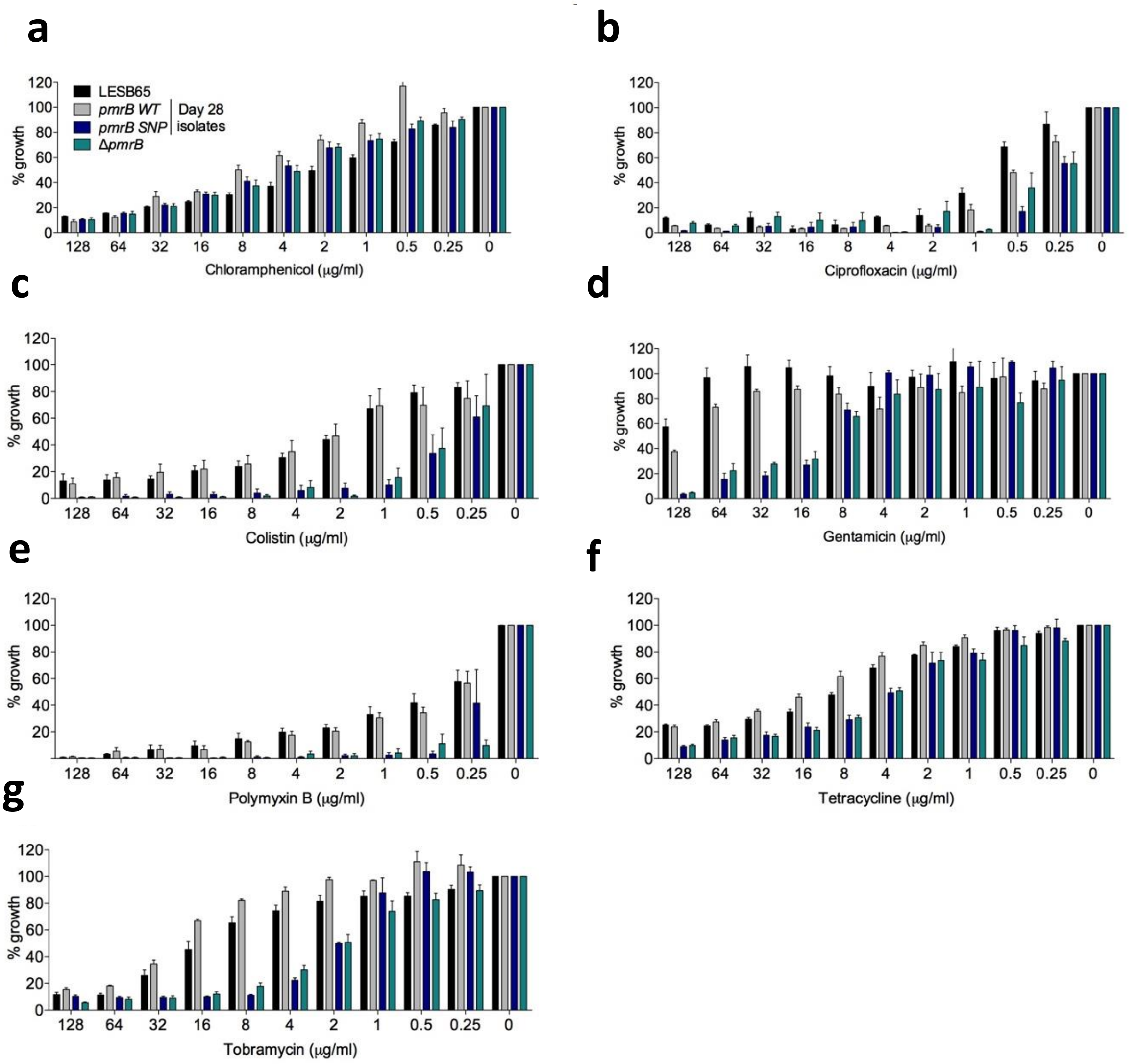

Supplementary Figure 2. Minimum inhibitory concentration assays. Percentage bacterial growth (OD_{600}) in the presence of antibiotic relative to growth in LB for **(a)** chloramphenicol, **(b)** ciprofloxacin, **(c)** colistin, **(d)** gentamicin, **(e)** polymyxin B, **(f)** tetracycline, and **(g)** tobramycin. Assays were performed at least five times with a minimum of three technical replicates per assay. Presented data are a composite of all assay results (mean ± SD). Significant differences vs LESB65 in two-way ANOVA: chloramphenicol (Δ*pmrB* $p<0.05$), ciprofloxacin (*pmrB* WT $p<0.001$, *pmrB* SNP $p<0.0001$, Δ*pmrB* $p<0.0001$), colistin (*pmrB* SNP $p<0.0001$, Δ*pmrB* $p<0.0001$), gentamicin (*pmrB* SNP $p<0.0001$, Δ*pmrB* $p<0.0001$), polymyxin B (*pmrB* SNP $p<0.0001$, Δ*pmrB* $p<0.0001$), tobramycin (*pmrB* SNP $p<0.0001$, Δ*pmrB* $p<0.0001$), tetracycline (*pmrB* SNP $p<0.005$, Δ*pmrB* $p<0.0001$).

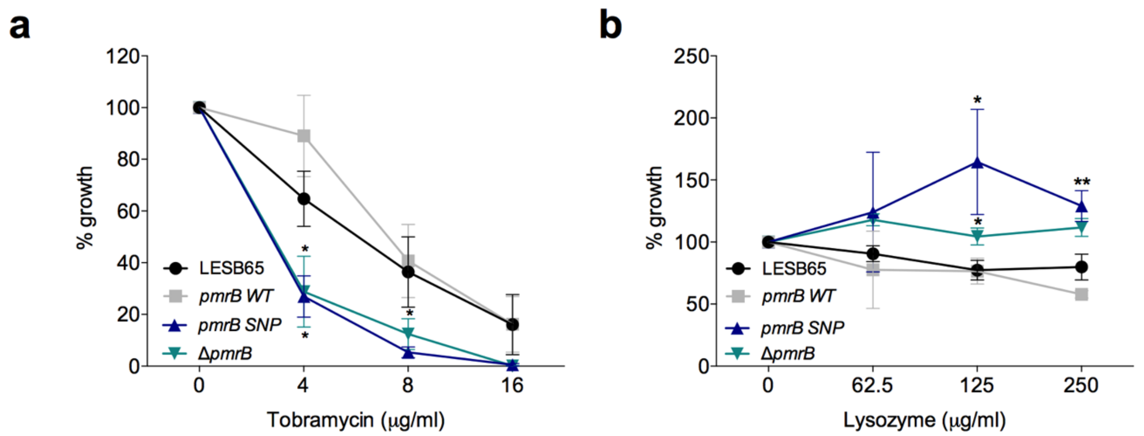

Supplementary Figure 3. The effect of *pmrB* mutation on *P. aeruginosa* grown in biofilm. (A) Percentage growth in tobramycin-containing artificial sputum media (ASM) relative to no-antibiotic control. **(B)** Effect of engineered, inhibition-resistant lysozyme on growth of *P. aeruginosa* in ASM relative to no-lysozyme control. Data presented are mean ± s.d. and are a composite of 3 independent experiments, each containing 3 technical replicates per isolate. *'s represent significant differences in two-way ANOVA with Dunnett's multiple comparison test versus LESB65. * = $p<0.05$, ** = $p<0.01$.

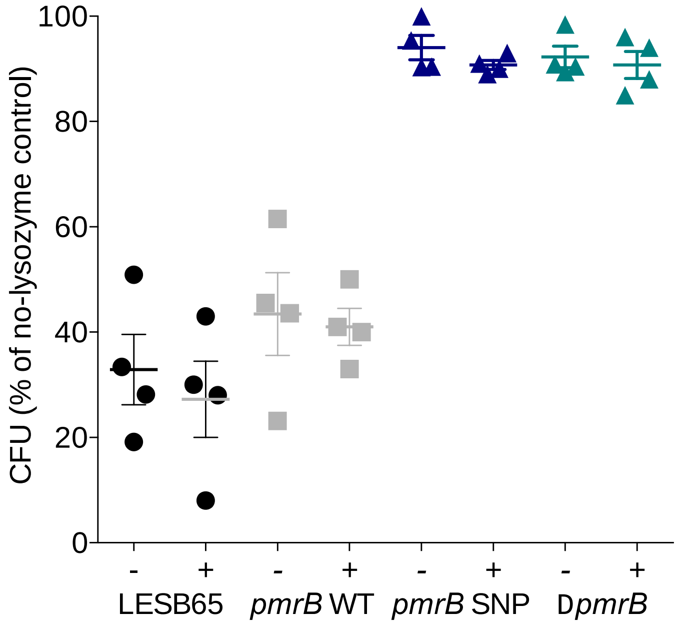

Supplementary Figure 4. Charge-engineering does not influence the susceptibility of *pmrB* mutants to lysozyme in planktonic culture. Isolates were grown in LB in the presence of 16 ug/ml native (-) or charge-engineered (+) lysozyme. Growth is presented as a percentage of the no-lysozyme control for each isolate and data points are individual biological replicates.

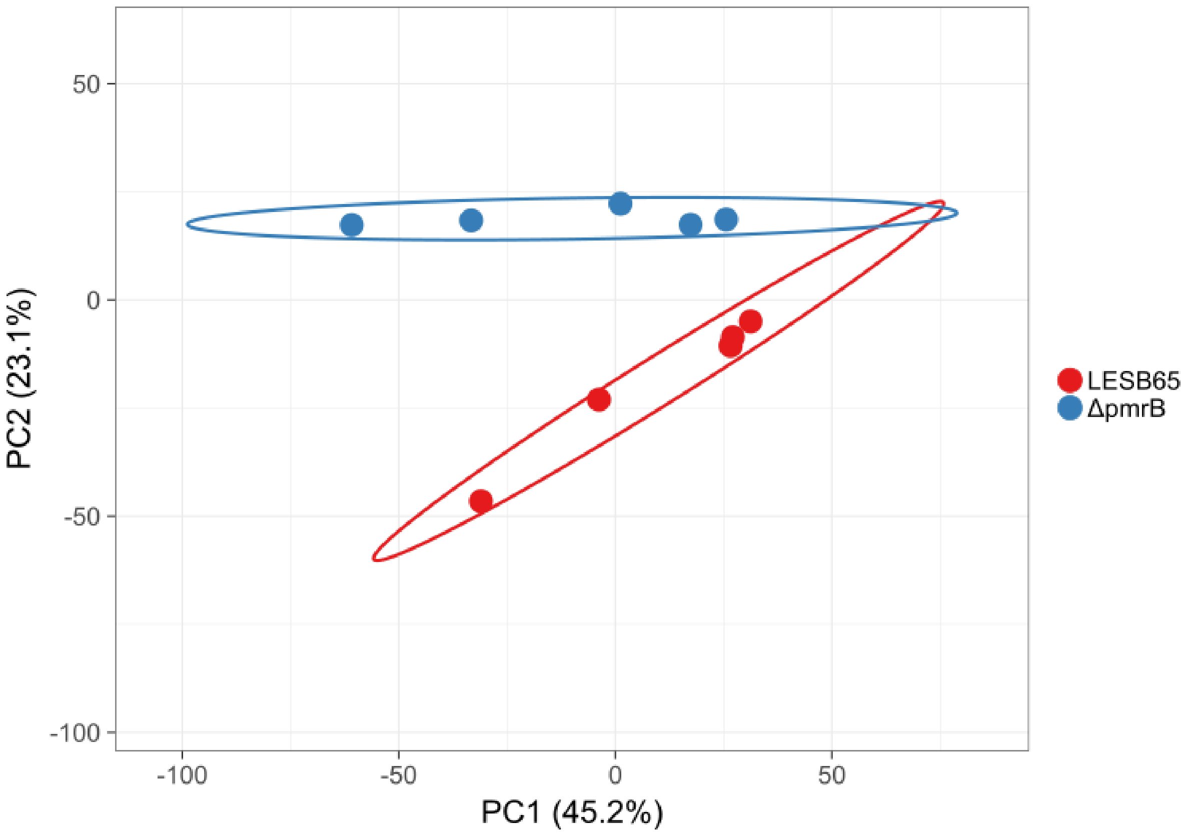

Supplementary Figure 5. Principal component biplot of LESB65 and Δ*pmrB* proteomics data showing all proteins detected. Five biological replicates were performed for each isolate.

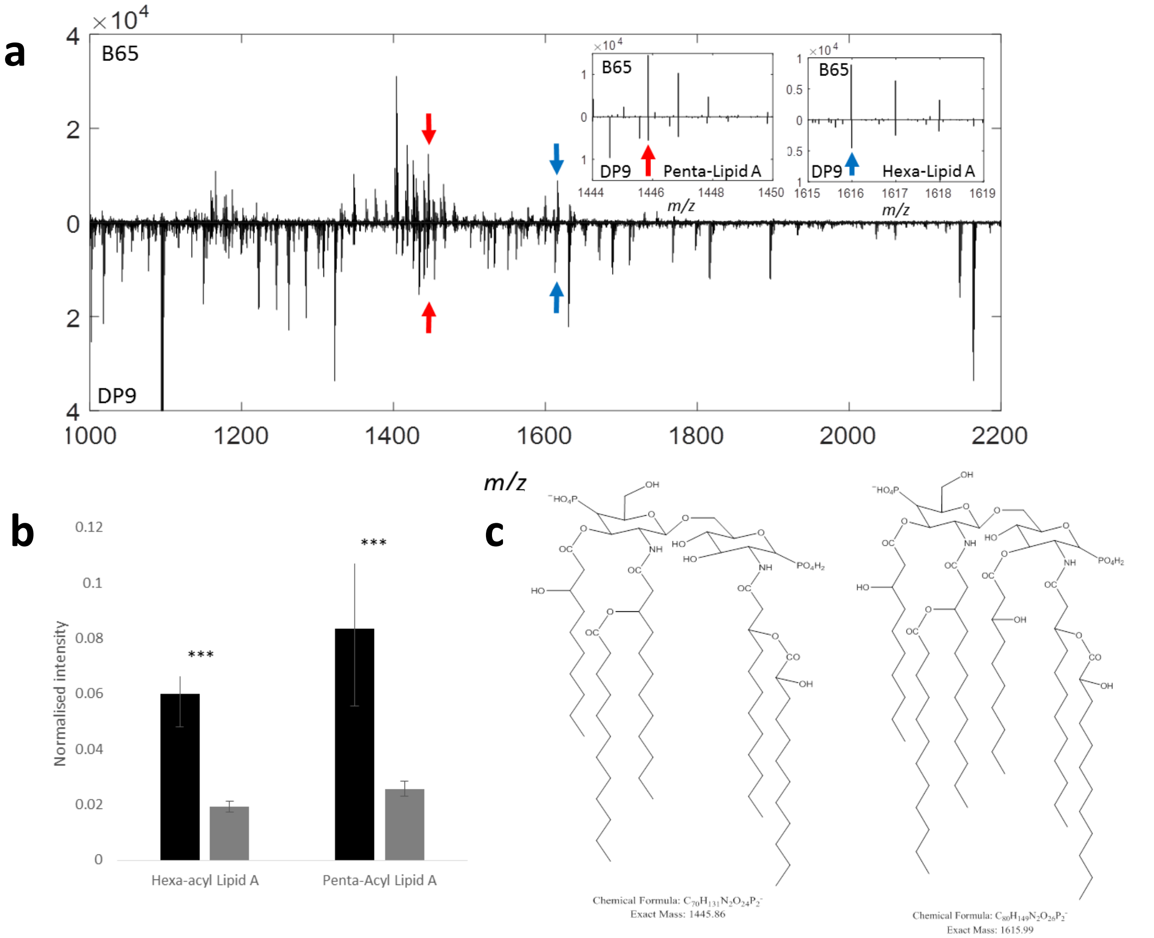

Supplementary Figure 6. Lipid A modifications in LESB65 and ΔpmrB. (a). Negative ion mode MALDI-TOF MS spectra of intact heat-inactivated LESB65 and ΔpmrB (m/z 1000 – 2200). Hexa–acyl lipid A (blue arrows) was measured at 1615.9873 (theoretical mass = 1615.9879, Δ ppm = - 0.37) and penta–acyl lipid A (red arrows) measured at 1445.8531 (theoretical mass = 1445.8572, Δ ppm = - 2.8). (b) Normalised intensity of measured hexa–acyl lipid A and penta–acyl lipid A of LESB65 (black) and ΔpmrB (grey). Four biological replicates (6 technical replicates) of each isolate, mean ± SD, *** = p<0.001 in unpaired t-test with Welch's correction. (c) Chemical structures of penta-acyl lipid A (left) and hexa-acyl lipid A (right).

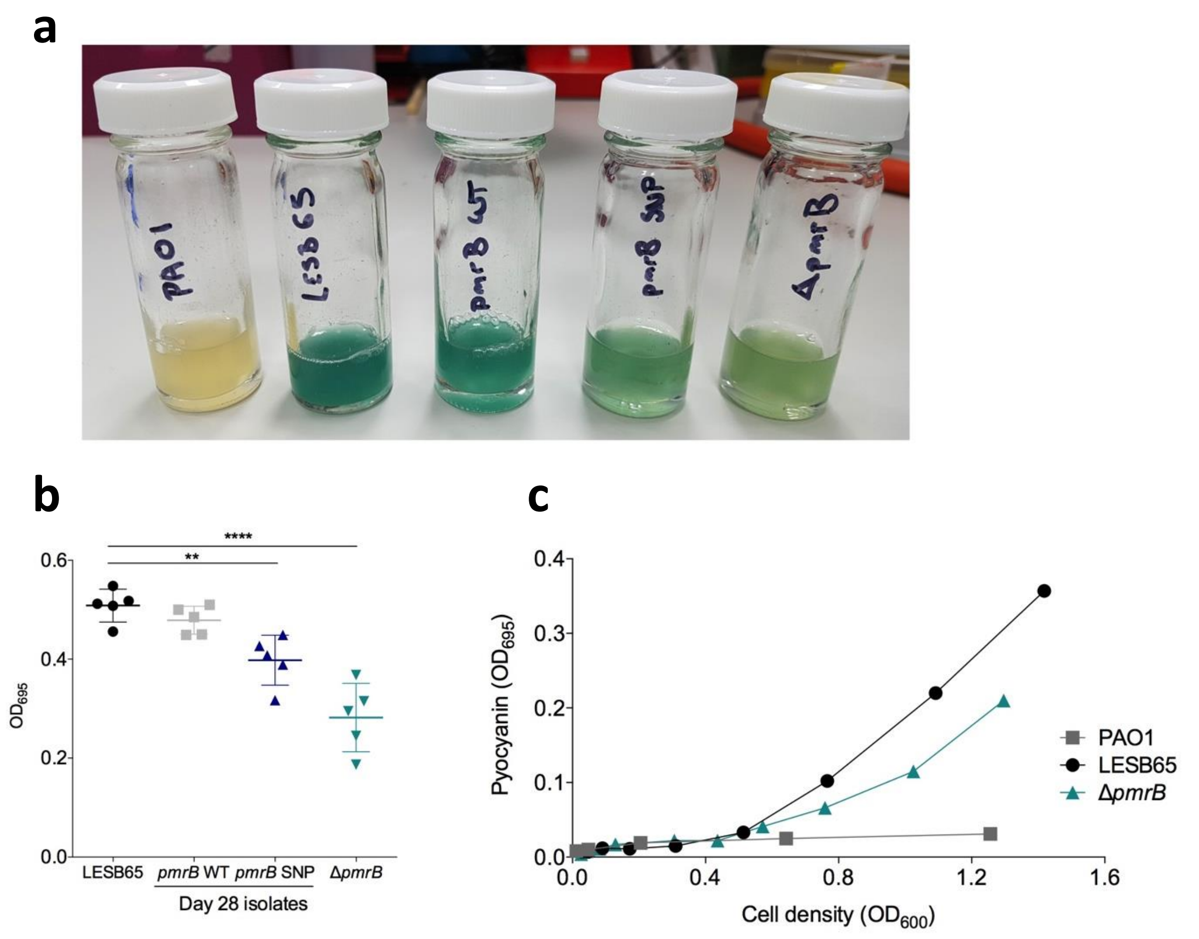

Supplementary Figure 7. Pyocyanin production by LESB65 and Δ*pmrB*. (a) Overnight cultures of *P. aeruginosa* PA01, LESB65, *pmrB* WT, *pmrB* SNP and Δ*pmrB* grown from a single colony. **(b)** Quantification of pyocyanin production by OD_{695} reading of cell-free culture supernatants from overnight growth. **(c)** Pyocyanin production (OD_{695}) versus cell density (OD_{600}) during 10 hours of growth following subculture of an overnight culture of a single colony. Each data point represents a single biological replicate. ** = $p<0.01$, **** = $p<0.0001$ in one-way ANOVA with Dunnett's multiple comparison.

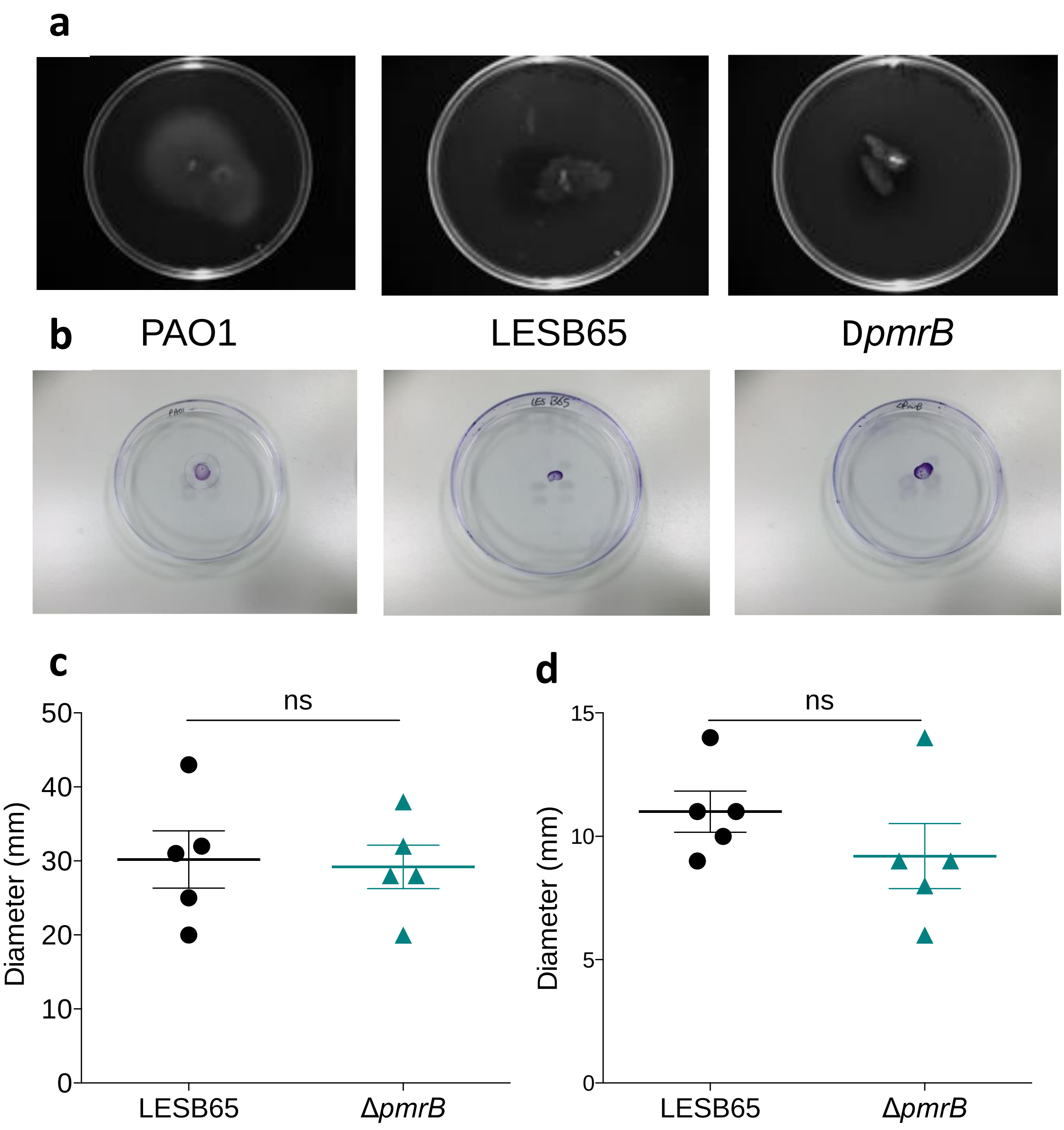

Supplementary Figure 8. Swimming and twitching motility are comparable in Δ*pmrB* and LESB65. (a) Swimming and **(b)** twitching motility of PAO1, LESB65 and Δ*pmrB* on/in agar. **(c)** Swim and **(d)** twitch diameter of LESB65 and Δ*pmrB* after 14 and 24 hour growth, respectively. Data points are individual biological replicates. ns = non-significant in unpaired t-test with Welch's correction.

Supplementary Table 1. Mutations identified by PacBio sequencing in *pmrB* SNP and *pmrB* WT. REF = reference (LESB65), ALT = alteration.

**Position**	**Gene**	**Annotation**	**REF**	**ALT**	***pmrB* SNP**	***pmrB* WT**
1461970	PALES_13561	hypothetical protein	C	T		Thr>Thr
1462067	PALES_13561	hypothetical protein	G	A		Val>Met
1462105	PALES_13561	hypothetical protein	T	C		His>His
1462122	PALES_13561	hypothetical protein	C	T		Thr>Ile
2108526	PALES_19541	Oxidoreductase	C	G	Gly>Gly	
2621560	PALES_24391	Nitric oxide reductase transcription regulator NorR2	G	T	Thr>Asn	
3326890	PALES_30151	TonB-dependent receptor	C	G		Leu>Leu
3326928	PALES_30151	TonB-dependent receptor	C	G		Thr>Ser
3496996	PALES_31711	Cardiolipin Synthase 2	G	C		Arg>Pro
3560685	PALES_32271	Putative Transmembrane Sensor	G	C	Ala>Gly	
3899297	PALES_35161	Putative Solute Binding Protein	C	G		Thr>Thr
4531964	PALES_41021	Glutathione-regulated Potassium-efflux system protein KefB	T	G	Thr>Pro	
5699445	PALES_51621	pmrB Two component regulator system signal sensor kinase	T	A	Leu>Gln	
6247828	PALES_56441	Putative integral membrane transport protein	C	G		Ala>Pro
6473649	PALES_58431	WbpY Glycosyltransferase	A	G	Pro>Pro	Pro>Pro
6601388	PALES_59651	Ribonuclease P protein component	C	T		Ala>Thr

Supplementary Table 2. Antimicrobial susceptibility of *P. aeruginosa* PAO1 with transposon insertions in *pmrB*. Minimum inhibitory concentration (µg/ml) required for 90% growth inhibition. Data are median values of n=3 biological replicates.

**Antibiotic**	**Isolate**	**MIC90 (µg/ml)**
Tobramycin	PAO1	2
	pmrBtransposon 1 (phoAwp06q1A09)	1
	pmrBtransposon 2 (lacZwp08q1F09)	1
Colisitin	PAO1	8
	pmrBtransposon 1 (phoAwp06q1A09)	2
	pmrBtransposon 2 (lacZwp08q1F09)	2

Supplementary Table 3. Cif and Ivy upregulation during planktonic growth in *pmrB* mutants. Fold change in gene expression compared to LESB65 determined by qPCR performed on cultures grown in LB media and normalised using reference gene *proC*. Similar trends were observed with normalisation to *rpoD.* Data are from three biological replicates per isolate, each biological replicate containing two technical replicates.

**Gene**	**LESB65**	***pmrB* WT**	***pmrB* SNP**	**∆*pmrB***
cif	1	2.36	4.90	8.58
ivy	1	0.28	4.64	1.57

Supplementary Table 4. Antimicrobial susceptibility of patients isolates

a

	Patient A					
	**Isolate**	**MIC50**	**MIC90**		**MIC50**	**MIC90**
Colistin	B38	2	>128	Tobramycin	16	64
	49194	**0.5**	**8**		**1**	**16**
	B60	2	128		4	128
Polymyxin B	B38	0.5	>128	Tetracycline	32	>128
	49194	**0.25**	**8**		**6**	**64**
	B60	0.5	>128		16	>128

b

	Patient B					
	**Isolate**	**MIC50**	**MIC90**		**MIC50**	**MIC90**
Colistin	B49	2	>128	Tobramycin	16	>128
	B40	**1**	**128**		16	>128
	B41	16	>128		4	128
	B109	8	>128		4	>128
	B64	2	>128		16	>128
	49137	**1**	**128**		**2**	**64**
	B65	2	>128		32	>128
Polymyxin B	B49	2	128	Tetracycline	8	>128
	B40	1	64		8	>128
	B41	1	64		8	>128
	B109	**0.5**	32		**4**	**64**

B64	**0.5**	64	8	128
49137	**0.5**	**16**	8	**64**
B65	1	64	16	>128

Minimum inhibitory concentration (µg per ml) required for **(a)** 50% or **(b)** 90% growth inhibition. Median MIC50 from a minimum of 5 replicates (range 5-7). The most sensitive isolates for each antibiotic are in bold and isolates with *pmrB* mutations are shaded grey.

Supplementary Table 5. Cif and Ivy upregulation during planktonic growth in clinical isolates with *pmrB* mutations. Fold change in gene expression compared to paired isolates from the same patient determined by qPCR performed on cultures grown in LB media and normalised using reference gene *proC*. Similar trends were observed with normalisation to *rpoD.* Data are from three biological replicates per isolate, each biological replicate containing two technical replicates.

	**Isolate**	***cif***	***ivy***
**Patient A**	B38	1	1
	49194 (*pmrB* mutant)	3.84	2.41
**Patient B**	B40	1	1
	49137 (pmrB mutant)	3.41	3.55
