# Supplementary material for: Evolutionary trade-offs associated with loss of PmrB function in host-adapted Pseudomonas aeruginosa
Source: Nat Commun. 2018 Jul 6;9:2635. doi: 10.1038/s41467-018-04996-x (PMC6035264; doi:10.1038/s41467-018-04996-x)
Supplement: Supplementary file 2 — Description of Additional Supplementary Information [file 41467_2018_4996_MOESM2_ESM.pdf]

Description of Additional Supplementary Files

File Name: Supplementary Data 1
Description: Full proteomic dataset for LESB65 and Δ*pmrB* during exponential growth in broth.

File Name: Supplementary Data 2
Description: Full proteomic dataset for LESB65, Δ*pmrB* and *pmrB* SNP during exponential growth in broth.

File Name: Supplementary Data 3
Description: ChemDraw file for the hexa-acylated LPS structure in Supplementary Figure 6.

File Name: Supplementary Data 4
Description: ChemDraw file for the penta-acylated LPS structure in Supplementary Figure 6.
